# Distinct Patterns of *PPARγ* Promoter Usage, Lipid Degradation Activity, and Gene Expression in Subcutaneous Adipose Tissue of Lean and Obese Swine

**DOI:** 10.3390/ijms19123892

**Published:** 2018-12-05

**Authors:** Bin Song, Shengwei Di, Shiquan Cui, Na Chen, Huan Wang, Xuan Wang, Qian Gao, Guizhi Tong, Hongbao Wang, Xuankai Huang, Liyan Ding, Ying Gao, Jun Liu, Xibiao Wang

**Affiliations:** 1 The Northeast Agricultural University, 59 Mucai St, Xiangfang District, Harbin 150030, China; songbin418@126.com (B.S.); dishengwei@163.com (S.D.); cococsq@163.com (S.C.); hxk87822@163.com (X.H.); 2 Animal Science Institute of Heilongjiang Province, 2 Heyi St, Longsha District, Qiqihaer 161005, China; guizhi315@163.com (G.T.); hongbao12345@sina.com (H.W.); dingliyan_1971@163.com (L.D.); 3 National Key Facility for Crop Resources and Genetic Improvement, Institute of Crop Science, Chinese Academy of Agricultural Sciences, Beijing 100081, China; chennvnv@163.com (N.C.); xuan.wang17@student.xjtlu.edu.cn (X.W.); gaoqian_dmb@163.com (Q.G.); gaoying@caas.cn (Y.G.); 4 Biotechnology Research Institute, Chinese Academy of Agricultural Sciences, Beijing 100081, China; wanghuan@caas.cn; 5 Department of Biological Sciences, Xian Jiaotong-Liverpool University, Suzhou 215123, China

**Keywords:** pig, subcutaneous adipose tissue, transcriptome, fatty acid degradation, PPARγ

## Abstract

Subcutaneous adipose tissue is a loose connective tissue specializing in the regulation of energy storage and metabolization. In domesticated pigs (*Sus scrofa*), the temporal development of subcutaneous adipose tissue is critical for meat production. However, the regulation of adipose tissue development remains unclear. Here, the subcutaneous adipose tissue development was characterized and compared in lean (Danish-Landrace) and obese (Min) pigs at juvenile and the juvenile-to-adult growth stages. Using RNA sequencing, we profiled the transcriptome of subcutaneous adipose tissue isolated from 4- and 16-week-old pigs and identified 24,718 expressed transcription units. Of them, 6327 genes were differentially expressed between the breeds and/or developmental stages. Compared with obese pigs, upregulated genes in lean pigs showed significant function and pathway enrichment in fatty acid degradation and mitochondrial functions. Further analysis uncovered the distinct usage preferences of the three alternative *peroxisome* *proliferator-activated* *receptor* *γ* (*PPARγ*) promoters associated with the development of subcutaneous adipose tissue in both breeds. Transcriptome analysis of subcutaneous adipose tissue in lean and obese pigs suggested that marker-assisted selection of fatty acid degradation and PPARγ signaling pathways could be important directions for future pork quality improvement and modern breeding.

## 1. Introduction

Pig (*Sus scrofa*, 2*n* = 38) is an important domesticated animal for meat production. Phylogenetic studies based on whole-genome sequences have indicated that the domestication of pigs originated in multiple locations across Southeast Asia around 3 to 4 million years ago [1,2,3,4]. According to the World Watch List of Domestic Animal Diversity, there are 118 indigenous pig breeds in China [5]. To improve pork quality, over the last few decades breeders have been making efforts to increase muscle yield and decrease fat [6]. For instance, Danish-Landrace (DL) and Large-Yorkshire breeds are widely grown lean pig breeds with high growth rates and a low percentage of body fat [7,8]. Similar to muscle and intramuscular fat, subcutaneous fat is also one of the important factors determining the deliciousness of pork meat. Subcutaneous fat is especially critical for the flavors of sausage, ham, and bacon. Subcutaneous fat contributes about 65–75% body fat in pigs. Moreover, recent studies uncovered that backfat thickness is highly associated with the carcass features, such as intramuscular fat content, meat color, tenderness, drip loss, cooking loss, and collagen proteins [9,10,11]. Thus, backfat thickness can be considered as an important value for measuring meat quality. Nowadays, the market demand for pork quality is diversifying, and consumer requirements for meat with high quality fat are also growing fast. The indigenous Min pig is a well-known obese breed in northeast China due to its high body fat percentage and strong resistance to cold and general diseases [12,13]. These lean and obese pig breeds serve as ideal models for studying the diversity of fat deposition between European commercial and Chinese indigenous pigs.

Adipose tissue is a central site of metabolic control and cell communication [14]. White and brown adipose tissue are main histological divisions of adipose tissue with distinct physiological characteristics and biological functions [15,16,17]. White adipose tissue serves as an energy storage reservoir, whereas brown is for energy dissipation and heat generation. During the past few decades, adipogenesis studies based on in vitro cell culture systems have revealed that cAMP inducers, glucocorticoid agonists, and insulin or insulin-like growth factor are major effectors triggering the conversion of fibroblasts into adipocyte-like cells. Initiation, differentiation, and termination of adipogenesis are mainly regulated by a transcriptional hierarchy. In particular, peroxisome proliferator-activated receptor γ (PPARγ), a nuclear hormone receptor and transcription factor, plays a central role in regulating adipose tissue development [18], and PPARγ-mediated coordination of multiple upstream signaling pathways with downstream genes is necessary for in vitro adipogenesis. The transcription activities of different *PPARγ* isoforms are promoted by multiple factors, such as Kruppel-like factors (KLF) 5/9/15, CCAAT-enhancer binding proteins α/β/δ, early B-cell factor 1, sterol regulatory element binding protein 1, zinc finger protein 423, nuclear factor 1, SMAD1/5/8, cAMP response element binding protein, early growth response 2 (also called KROX20), glucocorticoid receptor, and signal transducer and activator of transcription 5A/B and can be repressed by GATA binding factors 2/3 and KLF2 [19]. In addition, adipogenesis can be inhibited by multiple signaling pathways, such as canonical wingless/integrated signaling, retinoic acid, tumor necrosis factor α, hedgehog signaling, mitotic arrest deficient/transforming growth factor β, and hypoxia-inducible factor 1α [20].

In the present study, the subcutaneous adipose tissue transcriptome of two pig breeds with different fat accumulation patterns, lean (DL) and obese (Min), were profiled at juvenile and juvenile-to-adult growth stages. Differentially expressed genes between breeds and growth stages were identified. Gene Ontology and Kyoto Encyclopedia of Genes and Genomes enrichment analyses showed that the development- and breed-preferentially expressed genes presented specific functional preferences. Transcripts produced by three alternative *PPARγ* promoters were profiled to determine the differences in promoter usage between breeds. The results of the present study provide reference information for future investigation of the complex interactions of stimulatory and inhibitory signals in fat-storing cells.

## 2. Results

### 2.1. Genome-Wide Identification of the Expressed Genes in Subcutaneous Adipose Tissue

**Figure 1:**
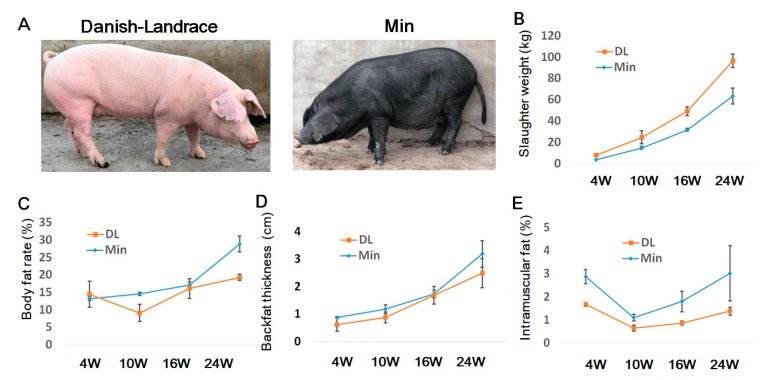
Developmental features of the Danish-Landrace (DL) and Min pigs. (**A**) DL and Min pigs at 24 weeks of age. (**B**–**E**) Slaughter weight, body fat rate content, backfat thickness, and intramuscular fat content of female DL and Min pigs at 4 weeks (4 W), 10 weeks (10 W), 16 weeks (16 W), and 24 weeks (24 W). Data presented with standard deviation bars per group (*n* = 3).

Male and female pigs of the lean breed Danish-Landrace (DL) and the obese breed Min were raised under the same environmental and nutritional conditions (Figure 1A, Table S1). We measured the backfat thickness from 4- to 24-week-old male and female pigs with five replicates using a real-time ultrasonography scanner. The backfat thickness showed a significant difference between the two breeds from 16 weeks but was not highly changed between the female and male pigs. Therefore, to simplify the experimental design, we used only female pigs in this study.

Next, we measured carcass traits of both breeds at 4, 10, 16, and 24 weeks of age (Figure 1B–E, Table S2). At 4 weeks, the slaughter weight, body fat content, and backfat thickness, intramuscular fat rate, mesenteric fat weight, perirenal fat weight, and greater omentum fat weight of both breeds were found to be similar. Most traits were clearly increased from 10 weeks to 24 weeks. At 16 weeks, the intramuscular fat rate, greater omentum fat of the Min pigs were significantly higher than the DL pigs and the backfat thickness of the Min pigs; body fat content and backfat thickness were slightly higher than those of DL, even though the slaughter weight of the Min pigs was lower. We speculated that the adipose tissues in Min and DL were systemically differentiating at 16 weeks and the developmental processes of subcutaneous adipose were distinct between the two breeds. Based on these results, we speculated that the 4-week (juvenile) and 16-week (juvenile-to-adult) age groups were the two to use for analysis of gene expression dynamics underlying subcutaneous adipose development.

**Figure 2:**
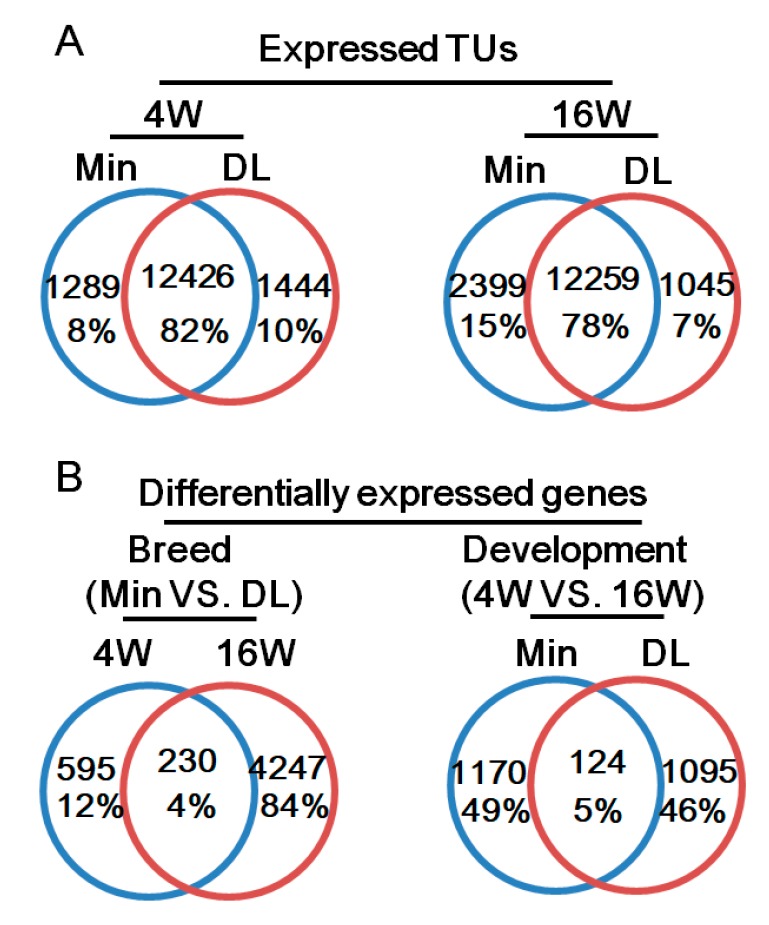
Gene identification and differentially expressed genes (DEGs) in DL and Min pigs. (**A**) The expressed transcription units in each pig breed. Transcription units with normalized read count were considered “expressed transcription units.” 4 W, 4-week-old pigs; 16 W, 16-week-old pigs. (**B**) DEGs in each pig breed (Fragments Per Kilobase of exon per Million fragments mapped (FPKM) fold-change > 2; *p* < 0.05).

The subcutaneous backfat samples of the two breeds were isolated from 4- and 16-week-old pigs to profile spatiotemporal changes of the subcutaneous adipose transcriptome using RNA sequencing (RNA-seq) with three biological replicates. RNA-seq reads were aligned to pig genome sequence release Sscrofa10.2 [21]. In total, 506.4 million sequencing reads were obtained, and 79.25% of them could be mapped to the genome (Table S3). Using our previous methods [22,23], the transcriptome was assembled, and Fragments Per Kilobase of exon per Million fragments mapped (FPKM) values and Transcripts Per kilobase Million (TPM) representing gene expression levels were normalized [24,25]. In total, 24,718 expressed transcription units (normalized read count >3 in at least one sample) were identified; about 78–82% were detected in both breeds (Figure 2A).

### 2.2. Spatiotemporal Expression of Subcutaneous Adipose-Associated Genes

Using DESeq2, differential expression analysis was performed with a FPKM fold-change > 2 and DESeq2 *p* < 0.05. There were 6327 significantly differentially expressed genes (DEGs) with clear breed- and/or age-based expression patterns. Of them, 5072 DEGs were differentially expressed between the breeds (Figure 2B). There were 4477 DEGs between the DL and Min breeds at 16 weeks, and 822 between the breeds at 4 weeks. Only 230 (3.6%) DEGs were shared between the breeds. Within each breed, there were 1294 and 1219 age-based DEGs in the Min and DL pigs, respectively; only 124 (5%) were shared between the breeds. These upregulated and downregulated genes are highlighted in Tables S4–S7. Of each comparison, 50% DEGs were upregulated in 16-week-old DL compared to those of 4-week-old DL; 73% were upregulated in 16-week Min compared to 4-week-old Min; 66% were upregulated in 4-week-old Min compared to 4-week-old DL; 24% were upregulated in 16-week-old Min compared to 16-week-old DL.

**Figure 3:**
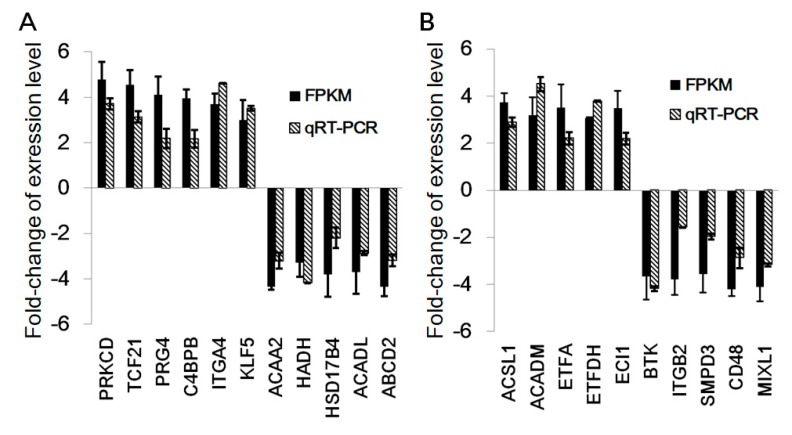
Experimental verification of gene expression level using qRT-PCR. (**A**) The relative expression levels (Min/DL) of 11 DEGs verified in 16-week-old pigs. (**B**) The relative expression levels (DL/Min) of 10 DEGs verified in 16-week-old pigs. Data presented with standard error bars per group (*n* = 3).

To verify the gene expression levels obtained by RNA-seq, quantitative real-time polymerase chain reaction (qRT-PCR) was used to determine the expression levels of the genes associated with subcutaneous adipose tissue development and fatty acid metabolism. qRT-PCR results were consistent with those of RNA-seq; expression fold-change values of the significant DEGs in the Min and DL pigs at 16 weeks are shown in Figure 3A,B, respectively. These results indicate that subcutaneous adipose tissues and their gene regulation networks are developmentally distinct between the breeds, suggesting different biological functions with age.

### 2.3. Functional Enrichment Analysis

**Figure 4:**
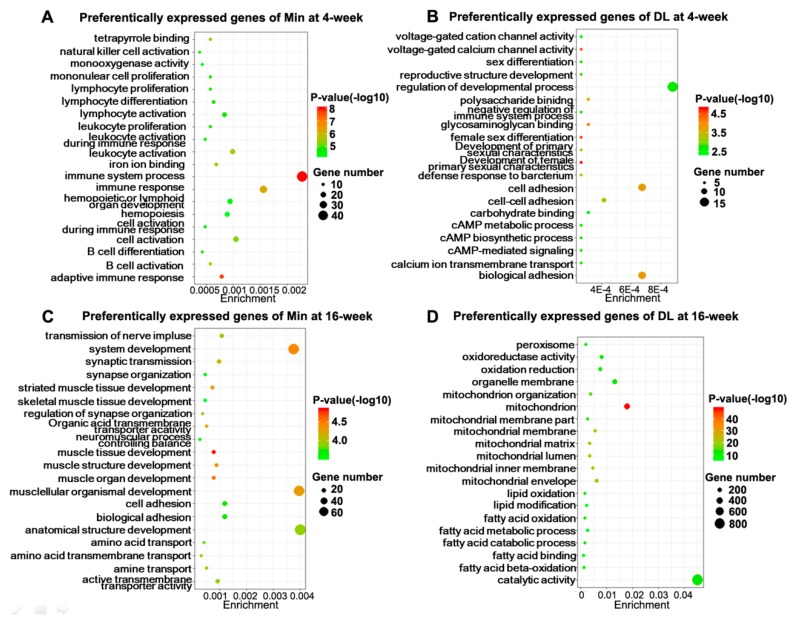
Gene Ontology (GO) enrichment of upregulated genes. GO enrichment of upregulated genes in 4-week-old Min (**A**), 4-week-old DL (**B**), 16-week-old Min (**C**), and 16-week-old DL (**D**) pigs. The X-axis provides the richness factor, which is calculated by dividing the upregulated gene number in a given GO term by the total gene number in the term of genome. The size and color of the bubbles represent gene number and enrichment significance according to hypergeometric testing, respectively.

To gain an overview on the functions of the DEGs, we carried out Gene Ontology (GO) enrichment analysis for the DEGs between the Min and DL pigs at the same developmental stage and identified 164, 186, 109, and 629 significantly enriched GO terms for upregulated DEGs in 4- and 16-week-old Min, as well as 4- and 16-week-old DL, respectively (Table S8–S11). Figure 4 shows the 20 representative GO terms selected from the top 30 most significantly enriched items of each group. At 4 weeks, GO terms included “adaptive immune response,” “leukocyte activation,” “B cell activation,” and several immune response-associated functions were significantly enriched in the upregulated DEGs of the Min pigs (Figure 4A); “sex differentiation,” “development of primary sexual characteristics,” “cell adhesion,” and “CAMP-mediated signaling” were significantly enriched in the upregulated DEGs of the DL pigs (Figure 4B). Of these DEGs, the expression levels of a subset of genes encoding proteins with known functions were significantly accumulated in the Min pigs, such as complement component 4 binding protein β [26,27,28], cluster of differentiation (CD) 1d [29], proteoglycan 4 [30,31], and regulatory proteins, including protein kinase Cδ, B cell-activating transcription factor, mix paired-like homeobox 1, γ-glutamyl transferase 1, transcription factor 21, protein tyrosine phosphatase, non-receptor type 6, signaling lymphocytic activation molecule family member 6, thymocyte-expressed molecule involved in selection 2, Fc fragment of immunoglobulin E receptor 1A, ectonucleotide pyrophosphatase/phosphodiesterase 1, ubiquitin-associated and SH3 domain-containing protein B, interleukin 1 receptor-like 1, Bruton’s tyrosine kinase, spleen-associated tyrosine kinase, integrin β2, sphingomyelin phosphodiesterase 3, hemoglobin subunit *ζ*, cannabinoid receptor 2, CD48, and integrin α4 (Table S4).

At 16 weeks, a subset of upregulated genes in the Min pigs was found to be significantly enriched in GO terms associated with muscle tissue development (Figure 4C), whereas those enriched in the DL pigs were mainly associated with fatty acid degradation and mitochondrial functions (Figure 4D). For example, ATP-binding cassette subfamily D member 2, acyl-CoA dehydrogenase, C-4 to C-12 straight chain, the α subunit of the mitochondrial tri-functional protein (HADHA), and acetyl-CoA acyltransferase-2 are key enzymes for the initiation, progression, and termination of fatty acid β-oxidation [32,33,34,35,36], while hydroxysteroid dehydrogenase 17-β 4, 2-hydroxyacyl-CoA lyase 1, and enoyl-CoA δ isomerase-1 are candidate genes pertaining to meat quality in pig breeding [37]. Thus, high expression of genes related to fatty acid metabolism should be considered an important molecular feature of lean pig breeds.

**Figure 5:**
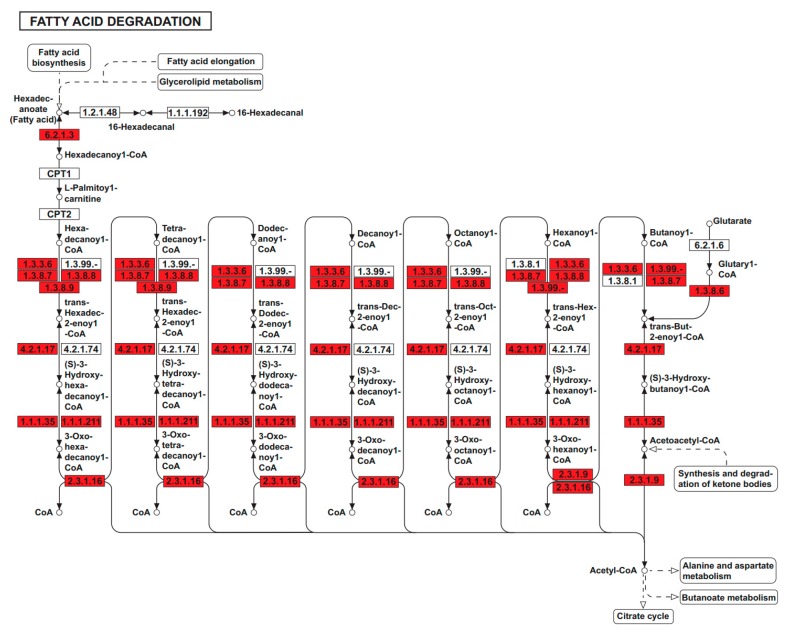
DL-specific DEG enrichment in the fatty acid degradation pathway. Enzymes encoded by upregulated genes in the DL pigs at 16 weeks are highlighted in red.

Kyoto Encyclopedia of Genes and Genomes (KEGG) enrichment analysis of the DEGs only obtained significantly enriched pathways for upregulated genes in 16-week-old DL pigs (Table S12). Of them, fatty acid degradation was one of the most significantly enriched items, and genes encoding catalytic enzymes in this pathway were globally upregulated in these pigs (Figure 5). This observation was consistent with GO enrichment results and morphological features of the DL pigs.

In summary, the functional enrichment analysis revealed some unexpected results: (1) A large number of the preferentially expressed genes in the Min and DL subcutaneous adipose tissue were not overlapped; (2) the enriched terms related to muscle development and the lipid metabolic pathway were observed in 16-week-old pigs, suggesting that the reduced fat levels of the lean pigs were likely the result of their relatively high ability of fatty acid metabolism; (3) the genes associated with the immune response were upregulated in the Min breed, which is consistent with the strong innate resistance of the Min to general diseases; (4) the genes related to sexual development were already highly expressed in the 4-week-old DL pigs indicating that these pigs were at the juvenile-to-adult growth stage, which provided molecular evidence for the regulation of sex hormones in the fast growth features of the DL. Furthermore, these genes found in subcutaneous adipose tissues could serve as biological markers for breeding selection.

### 2.4. Distinct PPARγ Promoter Usage Patterns Between Breeds

**Figure 6:**
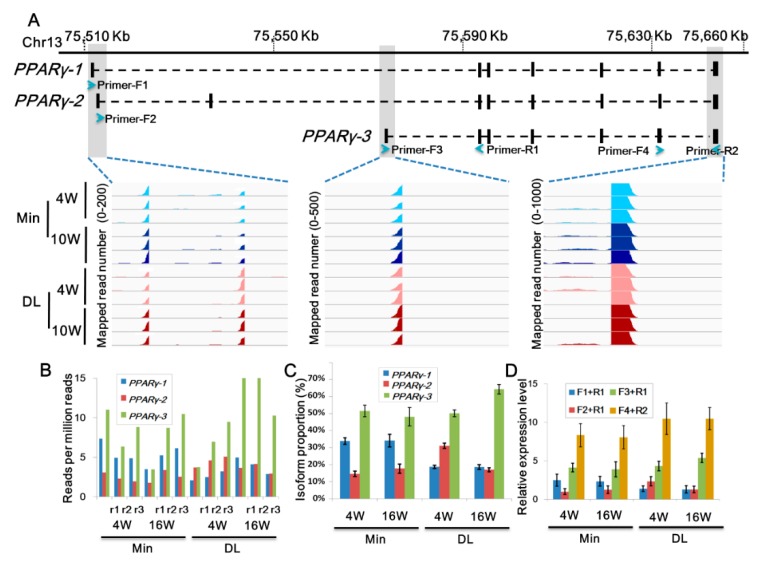
*PPARγ* promoter usage in the DL and Min pigs. (**A**) The assembled exon structures of *PPARγ* in pigs. Peaks show RNA-seq reads mapped to genomic regions in each pig breed at 4 weeks (4W) and 16 weeks (16W) of age (*n* = 3 biological replicates). (**B**) Read number mapped to the first exon of each PPARγ isoform. Read number was normalized by reads per million reads (RPM). (**C**) *PPARγ* promoter usages. PPARγ transcript isoform proportions were calculated by dividing the read number mapped to each first exon by the total read number mapped to all three first exons. (**D**) Expression level verification by qRT-PCR. Blue arrows in A give the exon regions used to design the qRT-PCR primers. Data presented with standard error bars per group (*n* = 3).

PPARγ is a master regulator in subcutaneous adipose tissue development. In our datasets, *KLF4*, *KLF5*, and several *PPARγ* pathway genes were differentially expressed between the two pig breeds (Table S7). However, *PPARγ* expression levels did not show significant changes between them suggesting that there is an unclear regulation process associated with the *PPARγ* locus. Using reference-based transcriptome assembly analysis, we characterized the exon structures of three *PPARγ* isoforms transcribed from three alternative transcription start sites (*PPARγ-1-3*; Figure 6). The first exon sequences of the three isoforms are different. Therefore, we could measure the transcript abundance of these exons by calculating the RNA-seq reads mapped to them, and using the mapped read count to quantitatively estimate the promoter activities of these isoforms (Figure 6A–C). We found that ~34.03% out of the *PPARγ* transcripts were generated from the *PPARγ-1* promoter in the Min breed at all the stages; whereas the average ratio from the DL *PPARγ-1* and Min *PPARγ-2* transcripts were 18.69% (*t*-test *p* < 4.77E-5) and 16.15% (*t*-test *p* < 5.22E-6), respectively. On the other hand, the DL pigs showed preferential usage of *PPARγ-2* and *PPARγ-3* promoters. At 4 weeks, 31.11% of all *PPARγ* transcripts derived from the *PPARγ-2* promoter in the DL pigs, which was significantly higher than the average *PPARγ-2* promoter usage ratio (16.20%, *t*-test *p* < 3.56E-4) in other samples. In 16-week-old DL pigs, 64.37% of all *PPARγ* transcripts were from the *PPARγ-3* promoter. In contrast, the average *PPARγ-3* promoter usage ratio was 49.83% in the Min pigs (*t*-test *p* < 1.67E-3). To further verify the promoter usage efficiencies in each breed, three pairs of sequence-specific primers were used to determine the expression levels of each transcript isoform (Figure 6D), and qRT-PCR results were consistent with those of RNA-seq. Taken together, these results demonstrate the different genotype- and age-specific usage patterns of the alternative *PPARγ* promoters, suggesting different putative functions during adipogenesis in pigs.

## 3. Discussion

Current gene annotation based on pig genome build 10.2 is composed of gene identification results from expressed sequence cDNA/tag sequences, bioinformatics prediction, and RNA-seq analyses. Several studies have carried out RNA-seq experiments to profile the transcriptomes of pigs [38,39,40,41]. The present study identified 24,718 expressed transcription units in the subcutaneous adipose tissue of lean (DL) and obese (Min) pig breeds at two different developmental stages (juvenile, 4 weeks old; juvenile-to-adult, 16 weeks old). Of them, 6327 showed breed- and/or age-based expression patterns. The assembled exon structures of these previously unannotated genes and their expression patterns indicated that most can be considered *bona fide* genes and that many genes remain to be identified in the pig genome, especially for those with complex gene structures and alternative splicing patterns. For example, the coding region of *PPARγ* spans more than 145 kb and encodes three transcript isoforms. The current transcriptome analysis characterized the exon structures of this gene and revealed different transcript isoform expression preferences for each pig breed and age. Thus, gene identification studies using more specific approaches, such as single-molecule transcript sequencing technology, in various tissues and genotypes are warranted in the future.

Pork meat samples are composed of mixed types of cells. It is technologically difficult to directly isolate intramuscular adipose tissue from meat samples. A previous study has profiled the transcriptome of skeletal muscle in pigs [13]. Now, with the marker genes and their expression patterns in subcutaneous adipose tissue presented in this study, we can expect the follow-up studies to compare and dissect the gene expression network of muscle, intramuscular fat, subcutaneous fat, and whole meat samples at multiple developmental stages.

Swine fat deposition in subcutaneous adipose tissue is determined by the balance between energy storage and usage [42]. Herein, genes associated with fatty acid degradation pathways were found to be systemically upregulated in DL versus Min pigs, including acyl-CoA dehydrogenase, C-4 to C-12 straight chain, *HADHA*, *HADH*, acetyl-CoA acyltransferase-2, hydroxysteroid dehydrogenase 17-β 4, 2-hydroxyacyl-CoA lyase 1, enoyl-CoA δ isomerase-1, ATP-binding cassette subfamily D member 2, electron transfer flavoprotein subunit α, peroxisome biogenesis factor 13, and electron transfer flavoprotein dehydrogenase. These results indicate that fatty acid degradation capability is an important factor distinguishing lean and obese pig breeds. In addition, a group of genes associated with muscle tissue development was found in the subcutaneous adipose tissue of Min pigs. Further studies need to be carried out to investigate the effect of fatty acid degradation on pork quality improvement and breeding selection, as well as to explore signaling crosstalk between subcutaneous adipose and muscle tissue during development.

In addition to its energy storage and metabolism functions, subcutaneous adipose tissue has also been known to play critical roles in immunity. Recent studies have indicated that centrally-situated subcutaneous adipose tissue is a major site for generating inflammatory responses and mediators as adipocytes in this area can set up communication with immune cells, such as T cells and macrophages, to mediate pathogen sensing and phagocytosis [43,44,45]. In the present study, a diverse subset of genes was found to be significantly differentially expressed in subcutaneous adipose tissue between lean and obese pigs, such as complement component 4 binding protein β, *CD1d*, and proteoglycan. The most significant functional enrichment of immune response-associated genes was found to be upregulated in the subcutaneous adipose tissue of 4-week-old Min pigs compared with that of DL. This suggests that the specific immune system of the Min has already been set up at the juvenile stage, which may confer the Min the strong innate resistance to general diseases at an early developmental stage. These results characterized the expression patterns of immune response-associated genes in subcutaneous adipose tissue and are helpful for better understanding genetic mechanisms associated with disease resistance and immunity in pigs.

PPARγ is an evolutionary conserved transcription factor belonging to the nuclear hormone receptor superfamily that plays important roles in adipogenesis and insulin sensitivity, type 2 diabetes, atherosclerosis, and cancer. In humans and mice, *PPARγ* has two major promoters and the *PPARg2* isoform plays a major role in their adipocyte differentiation [46,47,48,49]. In pigs, *PPARγ* encodes three protein isoforms that are produced by alternative promoters and alternative splicing at the 5′ end of each transcript. The one with seven exons, *PPARγ-3* in this study, showed similar sequence and splicing structures to the human *PPARg2* transcript. RNA-seq and isoform-specific qRT-PCR results indicate that the expression level of this isoform was much higher compared with the other two isoforms, suggesting that it may play major regulatory functions in subcutaneous adipocyte differentiation. The transcriptional activity of *PPARγ* alternative promoters are elaborately regulated by the specific DNA-binding proteins and epigenetic modifiers leading to the distinct expression patterns of the *PPARγ* isoforms. In the present analysis, *KLF4* [50], *KLF5* [51], and several *PPARγ* upstream regulatory genes were differentially expressed in lean and obese pigs. Thus, the distinct transcriptional activity and usage of the three *PPARγ* promoters in each pig breed can be considered a combined result that reflects that *PPARγ* and adipogenesis were regulated by the breed-specific cocktail effects composed of multiple upstream chemical and/or hormone signaling transduction pathways. However, more specific biological functions associated with each *PPARγ* isoform during adipogenesis remain to be clarified.

## 4. Materials and Methods

### 4.1. Animals and Ethics Statement

Animals used in the present study were conducted according to the Regulations for the Administration of Affairs Concerning Experimental Animals (Ministry of Science and Technology, China, June 2004) and approved by the Institutional Animal Care and Use Committee of Northeast Agricultural University (approved ID: CAS 235-2014, Harbin, China, 23 June 2014). Female DL and Min pigs were grown under the same environmental and nutritional conditions and housed in an environmentally- and dietary-controlled swine barn at the National Pig Breeding Farm in Lanxi, Heilongjiang Province, China. Animals were fasted 24-h prior to slaughter and slaughtered simultaneously. Backfat thickness between the third and fourth last ribs was measured using an HS1500 convex scanner (Honda Electronics, Toyohashi, Japan) with real-time B-mode ultrasonography.

### 4.2. Biological Materials and RNA-Seq Experiments

Subcutaneous backfat between the third and fourth last ribs was isolated from pigs and frozen immediately in liquid nitrogen prior to use. Total RNA was extracted using Trizol reagent and BAIXU^®^ TRNApure reagent with DNase I (Ambion^®^ TURBO, Austin, TE, USA) and purified using an RNeasy Mini Kit (Qiagen, Düsseldorf, Germany). Integrity of the RNA samples was determined using an Agilent Bioanalyser (Agilent, USA) with the cut-off RNA integrity score 8.5. The RNA sequence libraries were prepared with a BAIXU^®^ RNA Prep Pure Tissue Kit. The quality of cDNA libraries was checked using an Agilent 2200 TapeStation system (Agilent, Palo Alto, CA, USA). RNA libraries were sequenced using an Illumina^®^ HiSeq X10 (Illumina, San Diego, CA, USA) sequencing platform with a 150-nt pair-end sequencing protocol according to the manufacturer’s instructions. The fastq-formatted RNA-seq datasets were uploaded to the Genome Sequence Archive database (http://bigd.big.ac.cn/gsa/index.jsp, accessed on 5 December 2018) under accession no. CRA001170.4.3. RNA-Seq Analysis

The pig genome sequence (Sus10.2, GenBank ID: GCA_000003025.4) and annotation files were downloaded from the Ensemble database [21]. Raw RNA-seq sequences were aligned to the pig genome using HISAT2 and were assembled by cufflinks with reference genome annotation [52]. The RNA-seq count, FPKM, and TPM values of genes were calculated by HTseq-count and normalized using DESeq2. The differentially expressed genes were measured based on the DESeq2 results (*p* < 0.05, fold-change >2). A custom Perl script was used to integrate these results.

### 4.3. GO and KEGG Pathway and Functional Enrichment Analysis

GO and KEGG enrichment analyses were analyzed using AgriGO based on hypergeometric testing with Benjamini and Hochberg correction. All annotated pig genes with GO terms were considered as the entire population, and the DEGs were used as sampling values [53,54].

### 4.4. qRT-PCR

DNase I-treated RNA was reverse transcribed using SuperScript III and oligo-dT primers. cDNA was analyzed by qRT-PCR using SYBR Premix Ex Taq (Takara, Otsu, Shiga, Japan). All qRT-PCR reactions were performed for each cDNA sample with three replicates. Glyceraldehyde-3-phosphate dehydrogenase was used as an internal control. The primers used were designed by PRIME5 and their sequences are listed in Table S13.

## 5. Conclusions

In the present study, gene expression patterns in the subcutaneous adipose tissue of lean and obese pig breeds were characterized at two important developmental stages. Of the 24,718 expressed transcription units identified, 6327 were differentially expressed between each breed and/or developmental age. Functional analysis revealed that genes preferentially expressed in lean pigs were significantly enriched in fatty acid degradation and mitochondrial functions. In addition, expression levels of genes associated with fatty acid degradation were verified by qRT-PCR. Overall, the subcutaneous adipose tissue transcriptome profiled in lean and obese pig breeds in the present study highlighted genes in fatty acid degradation and PPARγ signaling pathways that could be important targets for future pork quality and marker-assisted modern breeding improvements.

## Supplementary Materials

Supplementary materials can be found at http://www.mdpi.com/1422-0067/19/12/3892/s1.
