# Supplementary material for: Distinct Patterns of PPARγ Promoter Usage, Lipid Degradation Activity, and Gene Expression in Subcutaneous Adipose Tissue of Lean and Obese Swine
Source: Int J Mol Sci. 2018 Dec 5;19(12):3892. doi: 10.3390/ijms19123892 (PMC6321263; doi:10.3390/ijms19123892)
Supplement: Supplementary file 1 [file ijms-19-03892-s001.zip › Supplemental Table 13. Real-time PCR validation of the DEGs analyzed by RNA-seq.docx]

Supplemental Table 11 Primers for Real-time PCR validation

| Gene | Primer Sequences (5'-3') | Product Size (bp) |
| --- | --- | --- |
| PRKCD | F: CCACCTCTTTTGCACCTTCC | 197 |
|  | R: CCAGCTTGAGGTCCCTGTAA |  |
| TCF21 | F: CTTCTCCAGGCTCAAGACCA | 185 |
|  | R: GTTTCCCGGCCACCATAAAG |  |
| PRG4 | F: AAATCCCCTGCTCTCACCTC | 204 |
|  | R: GGTGGGACTTTGCTTCTTCG |  |
| C4BPB | F: GGCCCTCCTGAGAATCCAAT | 182 |
|  | R:GTCTTGGGAGCTTCTGGGAT |  |
| ITGA4 | F: AATGGTCGAGCAGATGGGAT | 169 |
|  | R: ACACAGCAGAATCAGACCGA |  |
| KLF5 | F: CCCCAGCAGGCTACTTACTT | 176 |
|  | R: TGGGAATTAACTGGCAGGGT |  |
| ACAA2 | F: CACCCTGGAGCAGTTGAGTA | 174 |
|  | R: GACGCAAAATAGCCCACGAT |  |
| HADH | F: CTCGGCGAAGAAAATCCTCG | 192 |
|  | R: TCCGCAAACTTCTTCTTGGC |  |
| HSD17B4 | F:AGGGGACATGAAGGGAGTTG | 193 |
|  | R: ACGGTCCCTCAGAATTCCAG |  |
| ACADL | F: CTCCAGCTGCATGAAACGAA | 170 |
|  | R: TGAACTCGGGCATCCACATA |  |
| ABCD2 | F: GGGCATGGCTCGTATGTTTT | 216 |
|  | R: TCCAATTGTTCAAAGCGCCA |  |
| ACSL1 | F: TTTGTCCACGGAGAGAGCTT | 191 |
|  | R: AATGATTTGAGGCCAGCGTC |  |
| ACADM | F: CGAGTACCCTGTCCCACTAC | 154 |
|  | R: AGCAGTCTGAACCCCTGTAC |  |
| ETFA | F: CCCGAATAGCAGCCAAACTC | 187 |
|  | R: GCACTACCTCCACTTGTTGC |  |
| ETFDH | F: GATGAACAACCACGGCAACT | 198 |
|  | R: AATGTTGTCTTTGGTGCCCC |  |
| ECI1 | F: GCTCTCGGTGATAGCCAAGT | 154 |
|  | R: TTGGATGGCGTCTTTGGAGA |  |
| BTK | F: CTTCTTGAAGGAGCTGGGGA | 183 |
|  | R: CAGACACCATACAGCTGCAC |  |
| ITGB2 | F:GCCAAGTACAAGGTCAGCAC | 183 |
|  | R: CCTGGGATCCACGATGTCAT |  |
| GAPDH | F:CTACTCGGGCCTCTTCTGTG | 112 |
|  | R: GATTCTCCCGATCAGTCAGC |  |
| SMPD3 | F: TGCCAGAGGACAGTGACATT | 164 |
|  | R: ATCGGAGGAGCAGTTGTCAA |  |
| CD48 | F: GAATTCGCACTCTCTGGCAG | 163 |
|  | R: CAAGTCTGGCCCTGTCCTTA |  |
| MIXL1 | F: CGTCAGAGCGGCAAATTCTT | 173 |
|  | R: TGGGAGCTAGGGTCTGAGAT |  |
