# Supplementary material for: Distinct Patterns of PPARγ Promoter Usage, Lipid Degradation Activity, and Gene Expression in Subcutaneous Adipose Tissue of Lean and Obese Swine
Source: Int J Mol Sci. 2018 Dec 5;19(12):3892. doi: 10.3390/ijms19123892 (PMC6321263; doi:10.3390/ijms19123892)
Supplement: Supplementary file 1 [file ijms-19-03892-s001.zip › Supplemental Table 3. Genome-wide identification of the expressed genes in adipose tissue.docx]

Supplemental Table 1 Genome-wide identification of the expressed genes in adipose tissue

| Sample | Total read pair number | Left pair read  overall alignment rate | Right pair read  overall alignment rate |
| --- | --- | --- | --- |
| Min 4W r1 | 37795372 | 79.57% | 79.19% |
| Min 4W r2 | 41144225 | 77.49% | 77.13% |
| Min 4W r3 | 37521999 | 78.20% | 77.93% |
| Min 16W r1 | 55978334 | 81.05% | 81.05% |
| Min 16W r2 | 52038942 | 80.39% | 80.12% |
| Min 16W r3 | 48974443 | 80.62% | 80.33% |
| DL 4W r1 | 37298551 | 78.11% | 77.58% |
| DL 4W r 2 | 41694394 | 78.27% | 77.96% |
| DL 4W r 3 | 39401971 | 79.65% | 79.40% |
| DL 16W r1 | 33901617 | 80.27% | 80.00% |
| DL 16W r2 | 40133255 | 78.83% | 78.57% |
| DL 16W r3 | 40521234 | 78.56% | 78.30% |
